# Socioeconomic Vulnerability and Alcohol Relapse After Liver Transplantation for Alcohol-Associated Liver Disease: A Retrospective Study

**DOI:** 10.7759/cureus.111718

**Published:** 2026-06-29

**Authors:** Taha Shakarchi, Ban Al-Abayechi, Brian Quigley, Raza Malik

**Affiliations:** 1 Internal Medicine, Lahey Hospital and Medical Center, Burlington, USA; 2 Transplant Hepatology, Lahey Hospital and Medical Center, Burlington, USA; 3 Gastroenterology and Hepatology, Lahey Hospital and Medical Center, Burlington, USA

**Keywords:** alcohol-associated liver disease, alcohol relapse, employment status, liver transplantation, phosphatidylethanol, psychosocial assessment, sobriety duration, social vulnerability, transplant outcomes

## Abstract

Background

Alcohol relapse after liver transplantation for alcohol-associated liver disease is clinically consequential, yet the predictive value of commonly used pre-transplant psychosocial tools remains inconsistent. We evaluated pre-transplant psychosocial, socioeconomic, biomarker, and clinical variables associated with post-transplant alcohol relapse.

Methods

We performed a single-center retrospective cohort study of adults transplanted for alcohol-associated liver disease from January 2020 through February 2026. Relapse was defined as any post-transplant alcohol use by self-report or positive phosphatidylethanol (PEth) (or blood alcohol concentration (BAC) testing. Candidate predictors included employment status, age, Patient Health Questionnaire-9 (PHQ-9), Generalized Anxiety Disorder-7 (GAD-7), Stanford Integrated Psychosocial Assessment for Transplantation (SIPAT), CAGE score, Diagnostic and Statistical Manual of Mental Disorders, Fifth Edition (DSM-5) severity, support plan quality, pre-transplant phosphatidylethanol, sobriety duration, and substance use history. PHQ-9, GAD-7, and SIPAT values were harmonized into standard severity categories before analysis. Univariable testing used chi-square, Fisher's exact, and Mann-Whitney U tests. Logistic regression used complete cases for each predictor.

Results

Of 340 reviewed patients, 38 were excluded because their relapse status was indeterminate, leaving 302 patients. Twenty patients met relapse criteria; 13 of 20 events occurred beyond 12 months post-transplant. Employment status was associated with relapse: 14 of 120 unemployed patients relapsed, compared with 3 of 78 employed patients and 1 of 49 retired patients. Unemployment was associated with increased relapse odds (odds ratio (OR) 4.061, 95% confidence interval (CI) 1.297-12.714, p = 0.016). Relapsed patients were younger than non-relapsed patients, and age was inversely associated with relapse odds (OR 0.950 per year, 95% CI 0.909-0.993, p = 0.023). PHQ-9 showed a borderline, non-linear pattern, while SIPAT, sobriety duration, pre-transplant phosphatidylethanol, CAGE, DSM-5 severity, and support plan quality were not associated with relapse.

Conclusions

Unemployment and younger age were associated with post-transplant alcohol relapse, whereas several conventional pre-transplant tools did not stratify relapse risk. Employment status should not be used as an exclusion criterion but may identify socioeconomic vulnerability warranting enhanced post-transplant monitoring and support.

## Introduction

Alcohol-associated liver disease has become the leading indication for liver transplantation in the United States, reflecting both the declining burden of hepatitis C after direct-acting antiviral therapy and the rising impact of alcohol-related end-stage liver disease [1,2]. Although appropriately selected recipients can achieve favorable post-transplant outcomes, alcohol relapse remains clinically important because recurrent alcohol use may contribute to allograft injury, accelerated fibrosis, medication nonadherence, and worse long-term survival [3,4]. Sustained alcohol abstinence after transplantation can be challenging because relapse risk is influenced by addiction severity, psychosocial stressors, social support, and access to treatment.

Pre-transplant psychosocial assessment is therefore a central component of transplant evaluation. Programs commonly consider structured tools and variables such as the Stanford Integrated Psychosocial Assessment for Transplantation (SIPAT), CAGE, psychiatric diagnoses, sobriety duration, alcohol biomarkers, and social support. However, the predictive value of these tools has varied across studies, and rigid reliance on any single variable may incompletely characterize risk [5-8].

Contemporary transplant practice increasingly emphasizes individualized assessment and post-transplant support rather than fixed abstinence thresholds alone. In this context, socioeconomic vulnerability may be clinically relevant but underemphasized. We examined which pre-transplant psychosocial, socioeconomic, biomarker, and clinical variables were associated with post-transplant alcohol relapse in a single-center cohort of patients transplanted for alcohol-associated liver disease.

## Materials and methods

Study design and population

We conducted a single-center retrospective cohort study of adult patients who underwent liver transplantation for alcohol-associated liver disease at a single transplant center between January 2020 and February 2026. Inclusion required a primary transplant indication of alcohol-associated liver disease, including alcohol-associated cirrhosis or acute alcohol-associated hepatitis, confirmed at the time of transplant listing. Patients transplanted for other primary etiologies were excluded. Of 340 reviewed patients, 38 were excluded because post-transplant relapse status could not be determined from either self-report or objective testing, yielding a final analytic cohort of 302 patients. Follow-up duration was calculated from the date of liver transplantation to the most recent documented transplant clinic encounter, death, or the data abstraction date, whichever occurred first.

Relapse definition

Post-transplant alcohol relapse was defined as any documented alcohol use after transplantation, identified by self-report during clinical follow-up or by positive phosphatidylethanol (PEth) or blood alcohol concentration (BAC) testing at any post-transplant time point. Patients meeting either criterion were classified as relapsed. Post-transplant PEth testing was not performed at standardized routine intervals for all recipients. PEth testing was obtained when alcohol relapse was clinically suspected or when concerning clinical, laboratory, or psychosocial indicators prompted objective testing. This composite definition was selected to reduce underdetection from self-report alone, given the known limitations of self-disclosure in transplant and addiction care settings [9,10].

Variables

Pre-transplant psychosocial data were extracted from standardized transplant evaluation records. Candidate predictors included Patient Health Questionnaire-9 (PHQ-9) depression severity, Generalized Anxiety Disorder-7 (GAD-7) anxiety severity, SIPAT category, CAGE score, Diagnostic and Statistical Manual of Mental Disorders, Fifth Edition (DSM-5) severity, support plan quality, pre-transplant PEth testing, duration of sobriety before transplant, employment status classified as unemployed, employed, or retired, age at transplant, and other substance use history. SIPAT was obtained as part of the routine pre-transplant psychosocial evaluation performed by transplant social work personnel during standard transplant candidacy assessment. SIPAT values were abstracted retrospectively from the clinical record and were not administered prospectively for research purposes. PHQ-9, GAD-7, and SIPAT values were harmonized into standard severity categories before analysis when source data included a mixture of raw scores and category codes.

Statistical analysis

Categorical variables were compared using chi-square or Fisher's exact tests, as appropriate. Fisher's exact testing was used when expected cell counts were small. Age at transplant was compared using the Mann-Whitney U test. Complete-case univariable logistic regression was used to estimate odds ratios (ORs) and 95% confidence intervals (CIs) for selected predictors. Because the number of relapse events was low, multivariable modeling was exploratory, underpowered, and restricted to a maximum of three predictors. Adjusted estimates were interpreted as hypothesis-generating rather than confirmatory. A two-sided p-value less than 0.05 was considered statistically significant. Statistical analyses were performed using Python with SciPy and NumPy. This study was conducted under institutional review board oversight.

## Results

Cohort characteristics and relapse timing

The final cohort included 302 patients transplanted for alcohol-associated liver disease. Twenty patients (6.6%) met the composite relapse definition. Median post-transplant follow-up was 27.2 months (IQR 15.9-48.6; range 0.1-72.0 months). Relapse was most commonly identified beyond the first post-transplant year: 13 of 20 events (65%) occurred after 12 months. Relapsed patients were younger than non-relapsed patients (mean age 49.6 ± 8.6 vs. 55.0 ± 9.9 years, p = 0.010). Among the relapsed patients, 8 of the 20 (40%) were female.

Univariable associations

Employment status was associated with post-transplant alcohol relapse (χ² = 6.770, df = 2, p = 0.034). Among patients with known employment status, 14 of 120 unemployed patients relapsed (11.7%), compared with 3 of 78 employed patients (3.8%) and 1 of 49 retired patients (2.0%). PHQ-9 depression severity showed a borderline, non-linear association (χ² = 7.098, df = 3, p = 0.069); the highest relapse rate occurred in the severe depression category, whereas rates were lower in the mild and moderate categories. GAD-7 anxiety severity, DSM-5 diagnosis severity, pre-transplant PEth, length of sobriety at transplant, CAGE score, SIPAT category, support plan quality, and other substance use history were not associated with relapse. The full univariable associations and corresponding test statistics are summarized in Table 1.

**Table 1:** Univariable associations with post-transplant alcohol relapse (N = 302) †Age at transplant was analyzed using the Mann-Whitney U test. Categorical variables were analyzed using chi-square or Fisher's exact tests, as appropriate. Fisher's exact testing was used when expected cell counts were small. PHQ-9, GAD-7, and SIPAT values were harmonized to standard severity categories before analysis. Denominators vary because of missing data. CI, confidence interval; DSM-5, Diagnostic and Statistical Manual of Mental Disorders, Fifth Edition; GAD-7, Generalized Anxiety Disorder-7; PEth, phosphatidylethanol; PHQ-9, Patient Health Questionnaire-9; SIPAT, Stanford Integrated Psychosocial Assessment for Transplantation

Variable	N	Relapse n	Relapse %	Test	Statistic	p-value
Employment status	247	18	7.3%	Chi-square	χ²=6.770, df=2	0.034
Age at transplant ^†^	301	20	6.6%	Mann-Whitney U	U=1844.0	0.010
PHQ-9 depression severity	250	12	4.8%	Chi-square	χ²=7.098, df=3	0.069
GAD-7 anxiety severity	250	12	4.8%	Chi-square	χ²=4.882, df=3	0.181
DSM-5 diagnosis severity	267	18	6.7%	Chi-square	χ²=4.001, df=3	0.261
Pre-transplant PEth	302	20	6.6%	Chi-square	χ²=4.678, df=4	0.322
Length of sobriety at transplant	300	20	6.7%	Chi-square	χ²=3.152, df=5	0.677
CAGE score	131	3	2.3%	Chi-square/Fisher exact	χ²=1.793, df=4	0.774
SIPAT category	287	19	6.6%	Chi-square	χ²=1.374, df=2	0.503
Support plan quality	300	20	6.7%	Chi-square	χ²=0.510, df=4	0.973
Other substance use history	210	15	7.1%	Fisher exact	NA	1.000

Categorical relapse rates by employment status, PHQ-9, GAD-7, and SIPAT category are shown in Table 2.

**Table 2:** Categorical relapse rates by pre-transplant domain PHQ-9, GAD-7, and SIPAT categories reflect harmonized values. Missing data were excluded from each category-level denominator. SIPAT Good corresponds to scores 7-20; Minimally Acceptable 21-39; Poor 40-69. No patients with available SIPAT data were classified as High Risk. GAD-7, Generalized Anxiety Disorder-7; PHQ-9, Patient Health Questionnaire-9; SIPAT, Stanford Integrated Psychosocial Assessment for Transplantation

Domain	Category	N	Relapse n	Relapse %
Employment	Unemployed	120	14	11.70%
	Employed	78	3	3.80%
	Retired	49	1	2.00%
PHQ-9	None/minimal (<5)	83	2	2.40%
	Mild (5-9)	83	6	7.20%
	Moderate (10-19)	73	2	2.70%
	Severe (≥20)	11	2	18.20%
GAD-7	None/minimal (<5)	137	4	2.90%
	Mild (5-9)	63	4	6.30%
	Moderate (10-14)	27	1	3.70%
	Severe (≥15)	23	3	13.00%
SIPAT	Good (7-20)	60	2	3.30%
	Min. acceptable (21-39)	216	16	7.40%
	Poor (40-69)	11	1	9.10%

Logistic regression

**Figure 1:**
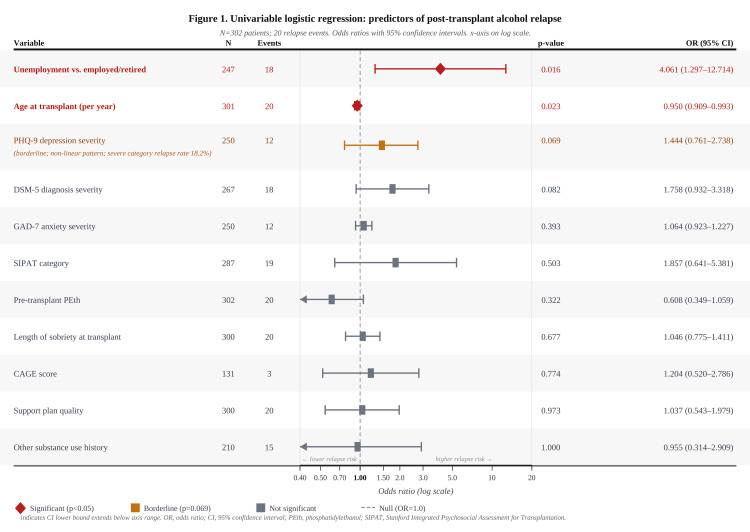
Univariable logistic regression for post-transplant alcohol relapse Univariable logistic regression odds ratios with 95% confidence intervals for post-transplant alcohol relapse. The x-axis is shown on a log scale. p-values correspond to univariable logistic regression models. Variables highlighted in red reached statistical significance; PHQ-9 showed a borderline association. CI, confidence interval; OR, odds ratio; PEth, phosphatidylethanol; SIPAT, Stanford Integrated Psychosocial Assessment for Transplantation

Complete-case univariable logistic regression showed increased relapse odds among unemployed patients compared with employed or retired patients (OR 4.061, 95% CI 1.297-12.714, p = 0.016). Older age at transplant was associated with lower relapse odds (OR 0.950 per year, 95% CI 0.909-0.993, p = 0.023), corresponding to approximately a 5% reduction in odds per additional year of age. In exploratory multivariable analysis, unemployment retained the largest adjusted effect size but did not reach statistical significance (adjusted OR 3.595, 95% CI 0.718-18.006, p = 0.120). These adjusted estimates should be interpreted cautiously because the exploratory multivariable model was underpowered, with a low events-to-predictor ratio and wide confidence intervals. The complete univariable logistic regression estimates are shown in Table 3 and Figure 1.

**Table 3:** Univariable logistic regression estimates for post-transplant alcohol relapse Complete-case univariable logistic regression models were used for each predictor. Odds ratios greater than 1 indicate higher odds of relapse, while odds ratios less than 1 indicate lower odds of relapse. CI, confidence interval; DSM-5, Diagnostic and Statistical Manual of Mental Disorders, Fifth Edition; GAD-7, Generalized Anxiety Disorder-7; OR, odds ratio; PEth, phosphatidylethanol; PHQ-9, Patient Health Questionnaire-9; SIPAT, Stanford Integrated Psychosocial Assessment for Transplantation

Variable	N	Events	OR	95% CI	p-value
Unemployed vs employed/retired	247	18	4.061	1.297-12.714	0.016
Age at transplant, per year	301	20	0.950	0.909-0.993	0.023
PHQ-9 category	250	12	1.444	0.761-2.738	0.069
DSM-5 diagnosis severity	267	18	1.758	0.932-3.318	0.082
GAD-7 anxiety severity	250	12	1.064	0.923-1.227	0.393
SIPAT category	287	19	1.857	0.641-5.381	0.503
Pre-transplant PEth	302	20	0.608	0.349-1.059	0.322
Length of sobriety at transplant	300	20	1.046	0.775-1.411	0.677
CAGE score	131	3	1.204	0.520-2.786	0.774
Support plan quality	300	20	1.037	0.543-1.979	0.973
Other substance use history	210	15	0.955	0.314-2.909	1.000

## Discussion

In this single-center cohort of 302 patients transplanted for alcohol-associated liver disease, unemployment and younger age were associated with post-transplant alcohol relapse, while several conventional pre-transplant evaluation variables did not stratify relapse risk, consistent with prior evidence showing variability in relapse predictors across transplant cohorts [3-8]. The observation that 65% of relapse events occurred beyond 12 months is clinically important because surveillance strategies concentrated only in the early post-transplant period may miss a substantial proportion of cases, consistent with prior work showing heterogeneous post-transplant alcohol use trajectories over time [8].

The association between unemployment and relapse should be interpreted carefully. Employment status is unlikely to represent a causal determinant in isolation. Rather, unemployment may reflect a broader cluster of socioeconomic vulnerabilities, including financial instability, reduced daily structure, impaired access to addiction treatment, limited social integration, weaker support networks, perceived stigma, and competing psychosocial stressors. The clinical implication is not that unemployed patients should be excluded from transplantation. Instead, unemployment may serve as a practical, readily available marker to identify recipients who would benefit from proactive social work engagement, addiction medicine follow-up, practical resource support, and longer post-transplant monitoring.

Younger age at transplant was also associated with relapse, consistent with prior data suggesting that younger recipients may have shorter duration of sobriety before end-stage disease, less stable social roles, or greater cumulative exposure to relapse risk [3,6,7]. Younger age is not modifiable, but it may inform the intensity and duration of post-transplant monitoring protocols.

Several null findings are equally informative. Stanford Integrated Psychosocial Assessment for Transplantation category, sobriety duration, pre-transplant phosphatidylethanol, CAGE, DSM-5 severity, and support plan quality were not associated with relapse in this selected transplant cohort. Prior studies have shown mixed associations between abstinence duration, psychiatric comorbidity, social support, and relapse, highlighting that these predictors may perform differently across cohorts and selection contexts [4-7]. These findings do not imply that psychosocial evaluation, sobriety assessment, or counseling engagement are without value. Rather, their discriminative performance may be attenuated after patients have already been selected through a rigorous transplant evaluation process in which counseling participation and multidisciplinary psychosocial review are expected. In this context, traditionally reassuring features, such as longer sobriety duration or engagement in counseling, may reflect baseline requirements for transplant candidacy rather than independent predictors of relapse after transplant. Importantly, our dataset could not measure the quality or effectiveness of the therapeutic alliance, patients’ acquisition of relapse-prevention skills, or their ability to re-engage with counseling during future stressors. These unmeasured dimensions may be clinically important and should be evaluated in future prospective studies.

Limitations of this study include its retrospective single-center design, modest event count, variability in post-transplant phosphatidylethanol monitoring frequency across the study period, missing employment data for 18% of the analytic cohort, and the composite relapse definition that includes any documented alcohol use regardless of severity or pattern. Post-transplant phosphatidylethanol testing was not performed routinely at standardized intervals for all recipients, although prior studies support phosphatidylethanol as an objective biomarker for detecting alcohol use in liver transplant populations [9,10]. Therefore, relapse events may have been under-ascertained, particularly among patients without self-reported use or clinical concern. The exploratory multivariable model was underpowered because of the limited number of relapse events, resulting in wide confidence intervals, and adjusted estimates should be interpreted as hypothesis-generating rather than confirmatory. Employment status was analyzed categorically and may not fully capture the spectrum of socioeconomic vulnerability, disability, caregiving, or other contextual factors.

Despite these limitations, these findings offer a clinically actionable message: post-transplant relapse risk assessment may need to extend beyond conventional scoring instruments and incorporate social vulnerability, younger age, and extended surveillance. Future prospective studies should standardize phosphatidylethanol monitoring schedules, distinguish isolated slips from sustained harmful relapse, and evaluate whether targeted post-transplant interventions reduce relapse rates among high-risk recipients.

## Conclusions

In this single-center retrospective cohort of patients transplanted for alcohol-associated liver disease, unemployment and younger age were associated with post-transplant alcohol relapse, while several conventional pre-transplant variables, including SIPAT category, sobriety duration, pre-transplant phosphatidylethanol, and support plan quality, did not stratify relapse risk. These findings should not be interpreted as support for excluding unemployed patients from transplantation. Rather, employment status may serve as a practical marker of socioeconomic vulnerability, psychosocial stress, and support needs.

Extended relapse surveillance and targeted post-transplant support may be particularly important for younger and unemployed recipients. Future prospective studies should examine not only whether patients engage in counseling, but also the quality of therapeutic alliance, relapse-prevention skill development, and ability to re-engage with support during periods of psychosocial stress.
